# Awareness and perceptions of patient and public involvement in rare disease research in Japan: the role of patient advocacy groups and family members

**DOI:** 10.1186/s40900-026-00955-9

**Published:** 2026-08-13

**Authors:** Saori Watanabe, Kaori Muto, Akiko Nagai, Hideki Yui, Yukitaka Kiya

**Affiliations:** 1 Department of Public Policy, The Institute of Medical Science, The University of Tokyo, 4-6-1 Shirokane-dai, Minato-ku, Tokyo, 108-8639 Japan; 2 Laboratory for Biomedical Ethics and Co-design, RIKEN Center for Integrative Medical Sciences, E219, 1-7-22 Suehiro-cho, Tsurumi-ku, Yokohama, Kanagawa 230-0045 Japan; 3 Department of Computational Biology and Medical Sciences, Graduate School of Frontier Sciences, The University of Tokyo, 4-6-1 Shirokane-dai, Minato-ku, Tokyo, 108-8639 Japan; 4 Center for Birth Cohort Studies, University of Yamanashi, 1110 Shimokawahigashi, Chuo City, Yamanashi 409-3898 Japan

**Keywords:** Patient and public involvement, Rare disease research, Patient advocacy groups, Patient and family perspectives, Japan

## Abstract

**Background:**

Patient and public involvement (PPI) in medical research has increasingly been incorporated into research policies and funding frameworks in Japan, but empirical evidence on how patients and family members perceive PPI remains limited. This is particularly important in rare disease research, where patient populations are small and involvement may depend on organised patient communities. This study explored awareness, experience, perceived benefits and barriers, and support needs regarding PPI among rare disease patients and family members in Japan.

**Methods:**

A cross-sectional online survey was conducted between December 2021 and April 2022 through two national rare disease patient network organisations. Adults with a rare disease or family members of such individuals were eligible. Of 218 responses, 212 met the eligibility criteria and were analysed. Subgroup comparisons used Pearson’s chi-squared test, with Fisher’s exact test when expected cell counts were below 5.

**Results:**

The sample comprised 101 patients (47.6%) and 111 family members (52.4%); 172 of 209 respondents (82.3%) reported prior participation in a patient advocacy group (PAG). Prior awareness of PPI was reported by 29.7% and prior experience by 20.8%. PAG participation was the only respondent characteristic examined that was associated with awareness (33.1% vs. 13.5%; *p* = 0.018). Nearly all respondents identified at least one perceived benefit of PPI, most commonly advancement of disease research (84.9%). Response patterns differed between PPI-aware and PPI-unaware respondents, and between patients and family members. Compared with patients, family members more often identified advancement of disease research and incorporation of patient and public perspectives as benefits, lack of knowledge about medical research as a barrier, and educational opportunities for patients and families as a needed form of support.

**Conclusions:**

Prior PPI awareness was limited, and PAG participation was the only respondent characteristic examined that was associated with awareness. Patients and family members showed somewhat different perceptions, with family members more often emphasising both the value of PPI and the need for educational support. Meaningful PPI in rare disease research requires not only formal promotion but also practical infrastructure, including public information, education, and support for PAGs, to make involvement feasible and sustainable.

**Supplementary Information:**

The online version contains supplementary material available at 10.1186/s40900-026-00955-9.

## Abstract (plain language summary)

**Why did we do this study?:**

When patients and families help shape medical research, the research is more likely to reflect what matters to them. This is called patient and public involvement, or PPI. In Japan, interest in PPI is growing, but little is known about how people affected by rare diseases understand it, or what support they need.

**What did we do?:**

A total of 212 people living with a rare disease or family members of someone with a rare disease completed an online survey. The survey asked about awareness and experience of PPI, its possible benefits and difficulties, and the support needed to promote it.

**What did we find?:**

Most respondents had not heard of PPI. However, people who had taken part in a patient advocacy group were more likely to know about it. Almost all respondents saw benefits of involvement, especially for advancing research into their disease. Family members were more likely than patients to see PPI as a way to advance research and incorporate patient and public perspectives. They were also more likely to view a lack of knowledge about medical research as a barrier.

**What does this mean?:**

Rare disease patients and families see value in PPI, but many are not yet familiar with it. Meaningful PPI needs practical support, including accessible information, learning opportunities, and stronger support for patient advocacy groups.

**Supplementary Information:**

The online version contains supplementary material available at 10.1186/s40900-026-00955-9.

## Background

Patient and public involvement (PPI) refers to research carried out with or by patients and members of the public, rather than solely about or for them [1]. Internationally, PPI has become widely promoted in health research, yet its implementation has been uneven both across countries and across research areas within them. The relevance, acceptability, and accountability of PPI in practice depend on context, resources, and the quality of relationships between researchers and patient and public contributors [2, 3]. PPI also draws on broader research ethics traditions that emphasise respect for participants and the communities in which research is conducted [4]. Recent decades have seen growing institutional support for PPI in many countries, though the pace and shape of this development have varied considerably [3].

In Japan, PPI has increasingly been incorporated into research policies and funding frameworks since the late 2010s, but it has developed through a relatively recent and uneven trajectory compared with countries where PPI has been formally embedded in publicly funded research for longer. The Japan Agency for Medical Research and Development (AMED) has played a central role, publishing, in 2019, a PPI Guidebook aimed primarily at researchers, together with subsequent learning resources [5–7]. These developments have helped establish PPI as a recognised term within Japanese health research, but awareness and implementation have progressed at very different rates across disease areas and research communities. Recent empirical work has begun to document this unevenness in specific fields. In allergy research, a national survey across multiple diseases has documented limited but growing awareness of PPI, with notable gaps between the views of researchers and those of patient organisations [8]. In hay fever research, a qualitative study of a mobile health project has shown how PPI can be implemented in practice through sustained dialogue between researchers and patient contributors [9]. In cancer genomics, survey work has also examined the expectations and concerns of patients, family members, and the public regarding whole-genome sequencing studies [10]. Taken together, these studies suggest that PPI in Japan is developing through a patchwork of disease-specific initiatives rather than through a single unified framework.

Legislation and formal procedures are important drivers of PPI internationally, and the Japanese case is distinctive in this respect. Japan has no legislation mandating PPI in research: research-governance instruments such as the Clinical Trials Act [11] and the ethical guidelines for medical research [12] regulate the conduct of research but do not address the involvement of patients or the public in its design or conduct. Instead, involvement is promoted through soft law and administrative guidance. The working definition currently in use was issued by AMED as a recommendation [5]; no official definition has been established by the government or the Ministry of Health, Labour and Welfare. Involvement has been taken up in the national basic plans adopted for particular disease areas. The Fourth Basic Plan for Cancer Control, adopted by Cabinet decision in 2023, includes the promotion of patient and public involvement as a stand-alone item for the first time and calls for research to be advanced with the involvement of patients and survivors [13]. No comparable provision applies to rare and intractable diseases: the basic policy adopted under the Act on Medical Care for Patients with Intractable Diseases provides for the collection of patient data and the dissemination of research findings to the public, but not for involving patients or the public in the research process itself [14]. Involvement has thus entered Japanese policy one disease area at a time, and rare diseases have so far been left out — the gap that the present study addresses.

The rare disease field occupies a distinctive position within this landscape [15]. Rare diseases are characterised by very small and often geographically dispersed patient populations, limited treatment options, diagnostic uncertainty, and a continuing reliance on research to improve diagnosis, treatment, and care [16]. These features make research infrastructures such as natural history studies, patient registries, and patient-centred outcome measures particularly important, while also increasing the value of patient, family, and patient organisation involvement across the research and therapeutic-development lifecycle [17–20]. In such settings, patients and family members are often closely connected to patient advocacy groups (PAGs), which may serve not only as support communities but also as interfaces linking affected families to the research system [21, 22]. Internationally, PAGs in rare disease research have been shown to contribute to study design, protocol review, recruitment, and broader research engagement, while recent reviews also highlight both the value of patient involvement and the practical challenges of sustaining it in this field [15, 21–23]. In Japan, initiatives such as the Commons Project and RUDY JAPAN have shown that meaningful stakeholder and patient involvement is feasible in rare disease research, while also demonstrating that such involvement depends on sustained infrastructures, trust-building, and close engagement with patient communities [24, 25]. At the same time, because the number of potential contributors is limited, involvement may become concentrated among a relatively small number of patients and family members, raising concerns about burden, accessibility, and sustainability [15, 22].

Patient and public involvement is valued because patients and family members bring experiential knowledge that can improve the relevance, quality, and legitimacy of research. This rationale is particularly salient in rare disease research, where small, dispersed populations and limited evidence make first-hand patient and family knowledge an especially important resource. In Japan, such involvement is now promoted under the specific term “PPI” in national funding and policy frameworks, so familiarity with the term has practical consequences for whether patients and families can recognise and access emerging opportunities. Our interest, however, is in involvement as a concept and practice rather than in the label itself; we therefore treat awareness of the term as one accessible, policy-relevant indicator, interpreted alongside self-reported experience, and we distinguish awareness of the term, understanding of the concept, and experience of involvement.

Despite this growing policy and research interest, how rare disease patients and family members themselves perceive PPI in Japan has received limited direct empirical attention [5, 24]. Existing discussions have tended to focus on policy promotion or on individual practical initiatives, with less emphasis on how patients and family members understand PPI, what benefits they associate with it, what barriers they perceive, and what forms of support they consider necessary [8–10, 24]. Empirical evidence from the perspectives of patients and family members is therefore needed to move beyond the formal promotion of PPI and to consider how involvement can be made meaningful and feasible in practice [24]. This study aimed to explore awareness of PPI, self-reported experience of involvement, perceived benefits and barriers, and support needs among rare disease patients and family members in Japan. Using data from an online survey conducted through national rare disease patient networks, we sought to describe how PPI is currently understood within this community and to examine whether perceptions differed according to respondent characteristics, including patient or family member status, prior awareness of PPI, and participation in PAGs.

## Methods

### Study design and setting

This study was a cross-sectional online survey conducted in Japan between December 2021 and April 2022. The survey was designed to explore awareness of PPI, self-reported experience of involvement, perceived benefits and barriers, and support needs among rare disease patients and family members.

### Participants and recruitment

Participants were recruited through two national networks representing the rare disease community in Japan: the Japan Patients Association and the Support Network for Nanbyo Children of Japan. Through these organisations, adults aged 18 years or older who were either living with a rare disease or family members of individuals with a rare disease were invited to participate in the survey.

Responses were collected anonymously using Questant, a web-based survey platform provided by Macromill, Inc. Eligibility was verified through a respondent characteristic item asking whether the respondent was a person living with a rare disease, a family member of such an individual, or someone with another relationship to the rare disease community (e.g., a supporter not personally affected by the condition); response numbers and exclusions are reported in the Results.

Because recruitment was conducted through national patient network organisations, the study population should be understood as reflecting the views of rare disease patients and family members who were reachable through such networks, rather than as a statistically representative sample of all rare disease patients and families in Japan.

### Questionnaire development and measures

The questionnaire was developed specifically for this study with reference to previous studies and guidance on PPI and related forms of patient and public engagement — including a practical rare disease initiative in Japan (RUDY JAPAN [25]), international reviews of patient engagement and its benefits and barriers [26, 27], and the Guidance for Reporting Involvement of Patients and the Public 2 (GRIPP2) checklist [28]. Because a central aim was to capture the basic awareness and perceptions of rare disease patients and family members, many of whom were not yet familiar with PPI, we deliberately kept the questionnaire simple and the wording accessible so that respondents with little prior knowledge could answer with ease. The draft questionnaire was developed by the authors and pre-tested by five external colleagues to improve clarity, wording, and structure.

Representatives of the two cooperating rare disease patient network organisations provided patient and public input during questionnaire development. They reviewed and pilot-tested the draft questionnaire by completing the survey and providing feedback on items that were unclear or that patients and family members might find difficult or distressing. Their input was used to revise the wording and structure of the questionnaire and the accompanying invitation materials before the survey was finalised. This process is reported in accordance with the GRIPP2 short form checklist (Additional file 1) [28].

The questionnaire comprised four domains: (1) awareness of PPI and prior experience of involvement, (2) perceived benefits of PPI, (3) perceived barriers to involvement, and (4) support needs for promoting PPI, together with items on respondent characteristics. Awareness of PPI was assessed by asking whether respondents had been aware of PPI before taking part in the survey. Self-reported experience of involvement was assessed by asking whether respondents had previously been involved in PPI in medical research or drug development. For items on perceived benefits, barriers, and support needs, respondents were asked to select all applicable options from predefined response categories. An N/A (“none of the above applies”) option was provided for respondents who did not consider any of the listed options applicable; this option should therefore be interpreted as indicating no applicable item among the predefined responses rather than the absence of benefits, barriers, or support needs.

Respondent characteristics included sex, age, educational attainment, and whether the respondent was a patient or a family member. In addition, because PAGs were considered potentially important intermediaries between rare disease communities and the research system, the questionnaire included an item on prior participation in PAG activities.

Because PPI is not yet widely institutionalised or socially familiar in Japan, the survey was designed to capture respondents’ awareness of the term and their perceptions of the idea and practice of PPI as presented in the questionnaire, rather than assuming a shared prior understanding of the concept. At the beginning of the PPI section of the questionnaire, before the item on prior awareness, all respondents were presented with a short explanation of PPI based on the definition and examples in the AMED PPI Guidebook [5], describing what PPI is, its purposes, and concrete examples of involvement. Accordingly, the awareness item was intended to capture whether respondents had encountered the term before the survey, whereas responses on perceived benefits, barriers, and support needs were provided after reading this common explanation. The full explanatory text and the questionnaire are provided in Additional file 2.

### Statistical analysis

Descriptive statistics were used to summarise respondent characteristics and survey responses. Awareness of PPI and self-reported experience of involvement were described using frequencies and percentages. To examine whether awareness of PPI differed according to respondent characteristics, we compared responses by sex, educational attainment, patient or family member status, and prior participation in PAG activities.

For items on perceived benefits, barriers, and support needs, we first described the overall distribution of responses. We then conducted subgroup comparisons to explore whether response patterns differed according to selected respondent characteristics, specifically prior awareness of PPI (PPI-aware vs. PPI-unaware) and patient or family member status.

Comparisons were performed using Pearson’s chi-squared test. Fisher’s exact test was applied when any expected cell count was below 5. Two-sided *p*-values are reported throughout, and *p* < 0.05 was considered statistically significant. Given the exploratory nature of the study and the modest sample size, multiple comparisons were not formally adjusted.

All analyses were conducted using SPSS version 30.0 (IBM Corp., Armonk, NY, USA).

## Ethical considerations

The survey was conducted anonymously and did not collect personally identifiable information. Before accessing the questionnaire, potential participants were provided with information on the purpose of the study, the voluntary nature of participation, and their right not to participate. Respondents indicated their informed consent by checking the consent box before proceeding to the questionnaire.

## Results

### Respondent characteristics

Of 218 responses initially received, six were excluded because those respondents did not identify as either patients with a rare disease or family members of such individuals (respondent characteristic item), leaving 212 valid responses (97.2%) from patients with rare diseases and their family members for analysis. Table 1 presents the demographic characteristics of respondents. The sample comprised 89 males (42.0%) and 123 females (58.0%), with a mean age of 51.9 years. Respondents were almost evenly split between patients (*n* = 101, 47.6%) and family members (*n* = 111, 52.4%). The distribution by sex differed between the two subgroups, with family members more often female than patients. Age distributions were broadly similar. Of 209 respondents who answered the item on PAG participation, 172 (82.3%) reported prior participation, reflecting the recruitment approach through national patient networks; the proportion was similar among patients (81.8%) and family members (82.7%); three respondents did not answer this item.

**Table 1:** Respondent characteristics

Characteristic	Total (*n* = 212)		Patient (*n* = 101)		Family member (*n* = 111)	
	*n*	%	*n*	%	*n*	%
**Sex**						
Male	89	42.0	55	54.5	34	30.6
Female	123	58.0	46	45.5	77	69.4
**Age (years)**						
< 20	1	0.5	0	0.0	1	0.9
20–29	7	3.3	6	5.9	1	0.9
30–39	21	9.9	9	8.9	12	10.8
40–49	63	29.7	27	26.7	36	32.4
50–59	60	28.3	24	23.8	36	32.4
60–69	45	21.2	28	27.7	17	15.3
≥ 70	15	7.1	7	6.9	8	7.2
Mean (years)	51.9		52.6		51.2	
**Education**						
Junior high school	5	2.4	4	4.0	1	0.9
Senior high school	34	16.0	15	14.9	19	17.1
Technical/vocational school	24	11.3	13	12.9	11	9.9
Junior college	31	14.6	12	11.9	19	17.1
University	96	45.3	48	47.5	48	43.2
Graduate school	22	10.4	9	8.9	13	11.7
**PAG participation**ᵃ						
Yes	172	82.3	81	81.8	91	82.7
No	37	17.7	18	18.2	19	17.3

Data are n (%) within each column unless otherwise specified. PAG, patient advocacy group. ᵃPercentages for PAG participation are based on the number of respondents who answered this item (n = 209: patient n = 99, family member n = 110); three respondents (two patients and one family member) did not answer

### Awareness and experience of PPI

Prior awareness of PPI was reported by 63 respondents (29.7%), whereas 149 (70.3%) reported no awareness before completing the survey. Overall, 44 respondents (20.8%) reported prior experience of involvement in PPI-related activities in medical research or drug development.

Table 2 shows the associations between respondent characteristics and prior awareness of PPI. Awareness of PPI did not differ significantly by sex (*p* = 0.456), educational attainment (*p* = 0.354), or respondent type (patient vs. family member; *p* = 0.134). Prior participation in PAG activities was the only respondent characteristic examined that was associated with PPI awareness: 33.1% of respondents with PAG participation reported being aware of PPI, compared with 13.5% of those without such participation (Pearson’s chi-squared test, *p* = 0.018).

**Table 2:** Awareness of PPI according to respondent characteristics and PAG participation

Characteristic	Aware *n* (%)	Not aware *n* (%)	Total *n*	*p*-value
**Sex**				
Male	24 (27.0)	65 (73.0)	89	
Female	39 (31.7)	84 (68.3)	123	0.456
**Education**				
University or aboveᵃ	32 (27.1)	86 (72.9)	118	
Below universityᵃ	31 (33.0)	63 (67.0)	94	0.354
**Respondent type**				
Patient	35 (34.7)	66 (65.3)	101	
Family member	28 (25.2)	83 (74.8)	111	0.134
**PAG participation**ᵇ				
Yes	57 (33.1)	115 (66.9)	172	
No	5 (13.5)	32 (86.5)	37	0.018

Data are n (%) within each subgroup. p-values are from Pearson’s chi-squared test or Fisher’s exact test, as appropriate, and are shown on the last row of each comparison group

ᵃ University or above includes university and graduate school (n = 118); below university includes all other educational categories (n = 94)

ᵇ Three respondents who did not answer the PAG participation question are excluded from that subgroup analysis (n = 209). PPI, patient and public involvement; PAG, patient advocacy group

Notably, 12 respondents (5.7%) reported prior experience of involvement despite indicating that they had not been aware of PPI before the survey. This pattern suggests that some respondents may have engaged in activities broadly consistent with PPI in practice without recognising the term itself. This discrepancy between awareness of PPI terminology and self-reported experience of involvement is considered further in the Discussion.

### Perceived benefits of PPI

**Fig. 1:**
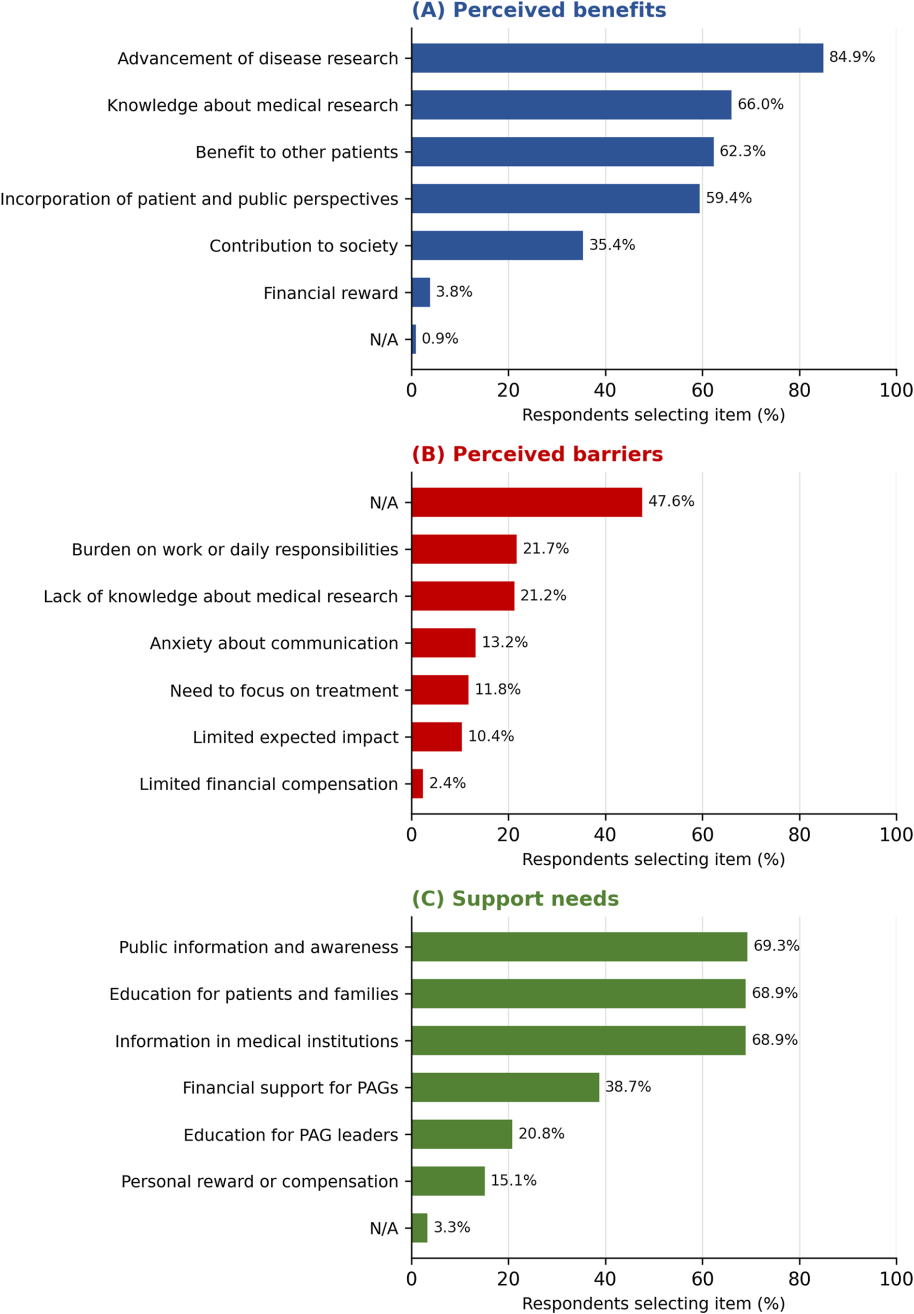
Perceived benefits, barriers, and support needs for PPI. Panel **A **shows perceived benefits of PPI, Panel **B **shows perceived barriers to involvement in PPI, and Panel **C **shows support needs for promoting PPI. Respondents (n = 212) could select multiple items in each domain. Bars show the percentage of respondents selecting each item. Colours denote domain (benefits, blue; barriers, red; support needs, green), consistent with Fig. 2. Item labels in the figure are abbreviated; full wording is provided in Additional file 2 (Survey Questionnaire). N/A indicates that none of the listed options applied. PPI, patient and public involvement; PAG, patient advocacy group

Figure 1A presents respondents’ perceptions of the benefits of PPI. Across the total sample (*n* = 212), the most frequently selected benefit was advancement of disease research (84.9%), followed by knowledge about medical research (66.0%), benefit to other patients (62.3%), and incorporation of patient and public perspectives (59.4%). Contribution to society was selected by 35.4% of respondents, while financial reward was rarely cited (3.8%). Only 0.9% of respondents selected N/A, indicating that nearly all respondents were able to identify at least one perceived benefit.

**Fig. 2:**
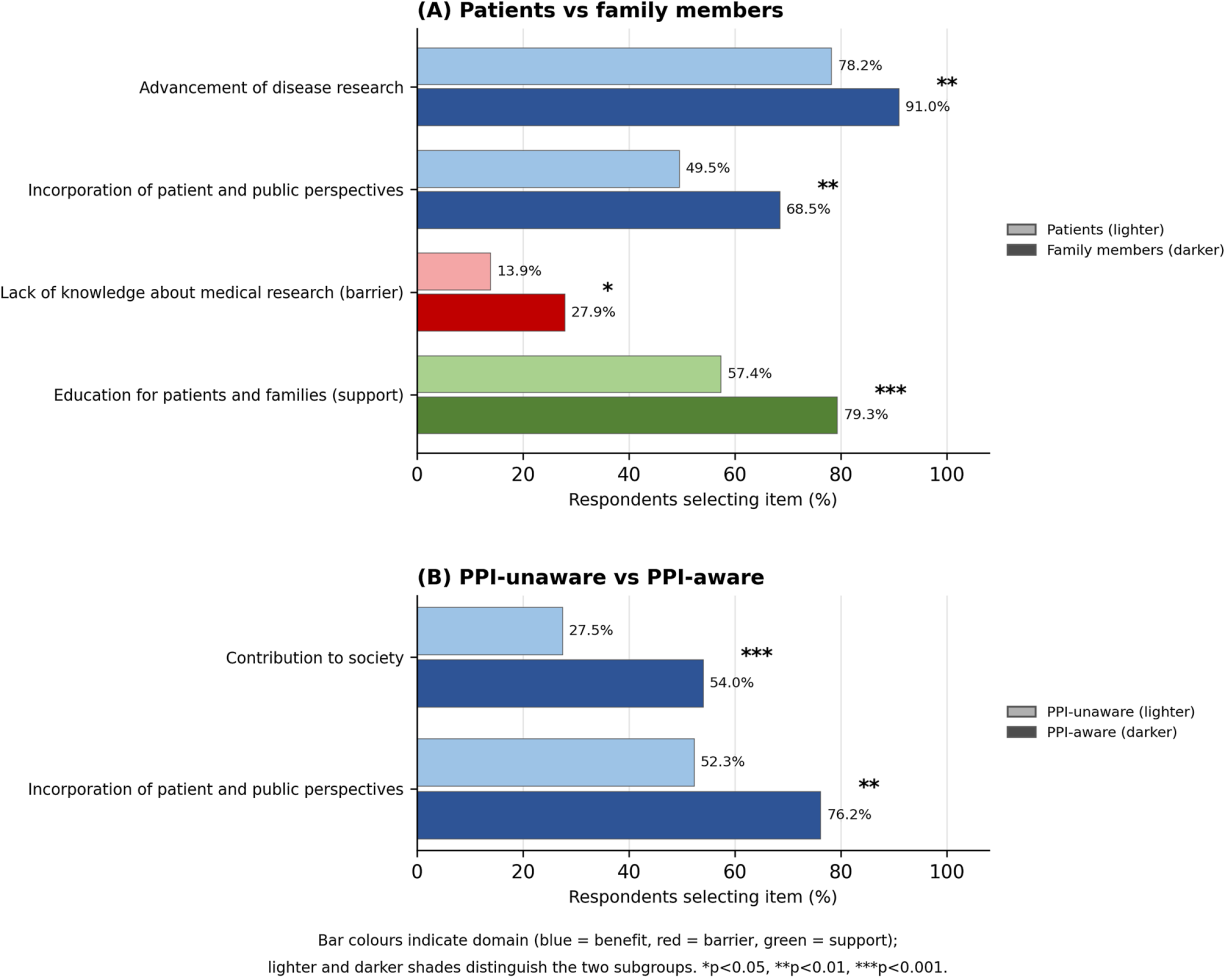
Exploratory subgroup comparisons of perceptions of PPI. Panel **A **shows differences between patients and family members, and Panel **B** shows differences between PPI-unaware and PPI-aware respondents. Only items showing statistically significant subgroup differences are presented. Bars show the percentage of respondents within each subgroup selecting each item. Colours denote domain (benefits, blue; barriers, red; support needs, green), consistent with Fig. 1. Asterisks indicate statistical significance by Pearson’s chi-squared test: *p < 0.05, **p < 0.01, ***p < 0.001. These subgroup comparisons were exploratory and were not adjusted for multiple comparisons. In both panels, every item shown was selected more frequently by the second subgroup named (family members in Panel **A**; PPI-aware respondents in Panel **B**). PPI, patient and public involvement

Response patterns differed between patients and family members (Fig. 2A). Family members more often selected advancement of disease research (91.0% [101/111] vs. 78.2% [79/101] among patients; *p* = 0.009) and incorporation of patient and public perspectives (68.5% [76/111] vs. 49.5% [50/101]; *p* = 0.005). Responses to the remaining benefit items did not differ substantially between the two groups.

Patterns also differed in some respects between PPI-aware and PPI-unaware respondents (Fig. 2B). PPI-aware respondents more often selected contribution to society (54.0% [34/63] vs. 27.5% [41/149] among PPI-unaware respondents; *p* < 0.001) and incorporation of patient and public perspectives (76.2% [48/63] vs. 52.3% [78/149]; *p* = 0.001). Responses to the remaining benefit items were broadly similar between the two groups.

### Perceived barriers to PPI

Figure 1B presents respondents’ perceptions of barriers to involvement in PPI. The most frequently selected response was N/A (47.6%), indicating that nearly half of respondents did not identify a specific barrier from the options provided. Among the listed barriers, the most frequently selected were burden on work or daily responsibilities (21.7%) and lack of knowledge about medical research (21.2%), followed by anxiety about communication with professionals (13.2%), need to focus on treatment or health management (11.8%), and limited expected impact on research (10.4%). Limited financial compensation was selected by only a small minority of respondents (2.4%).

No respondent left this item unanswered; N/A (“none of the above applies”) was an explicit option, selected by 101 respondents (47.6%). The proportion selecting N/A did not differ significantly between PPI-aware (42.9% [27/63]) and PPI-unaware (49.7% [74/149]) respondents (*p* = 0.364), providing no evidence that N/A selection was concentrated among those who were unaware of PPI.

Responses to the barrier items were broadly similar between PPI-aware and PPI-unaware respondents. Some variation was observed between patients and family members (Fig. 2A): family members somewhat more often identified lack of knowledge about medical research as a barrier (27.9% [31/111] vs. 13.9% [14/101] among patients; *p* = 0.012). Differences between patients and family members were not apparent for the other barrier items.

### Support needs for promoting PPI

Figure 1C presents respondents’ views on the types of support needed to promote PPI. The three most frequently selected items across the total sample were public information and awareness (69.3%), educational opportunities for patients and families (68.9%), and information in medical institutions (68.9%). Financial support for PAGs was selected by 38.7% of respondents, while educational opportunities for PAG leaders (20.8%) and personal reward or compensation (15.1%) were selected less frequently. Only 3.3% selected N/A, indicating that almost all respondents were able to identify at least one support need.

Responses to the support items were broadly similar between PPI-aware and PPI-unaware respondents. A difference was observed between patients and family members: family members more often identified educational opportunities for patients and families as a needed form of support (79.3% [88/111] vs. 57.4% [58/101] among patients; *p* < 0.001; Fig. 2A). This pattern is consistent with the other differences observed between patients and family members, as discussed below. Responses to the remaining support items were broadly similar across subgroups.

## Discussion

### Principal findings

This study explored how rare disease patients and family members in Japan perceive PPI. Across the sample, prior awareness of the term PPI was limited, even among respondents recruited through national rare disease patient networks, although a substantial minority reported some prior experience of involvement. Participation in PAG activities was the only respondent characteristic examined that was clearly associated with PPI awareness, whereas awareness did not vary appreciably by sex, educational attainment, or patient or family member status. Respondents generally recognised the value of PPI — most frequently for advancing disease research — while also pointing to educational opportunities, public information, and information in medical institutions as important forms of support. PPI-aware respondents and family members tended to endorse somewhat different aspects of PPI from PPI-unaware respondents and patients, respectively; the differences between patients and family members appeared consistently across benefits, barriers, and support needs, whereas those between PPI-aware and PPI-unaware respondents were largely confined to perceived benefits. Taken together, these findings suggest that PPI awareness and expectations in rare disease communities are shaped not only by individual characteristics but also by connections to organised patient communities and by respondents’ positions as patients or family members. The coexistence of limited terminological awareness and self-reported involvement not recognised under the PPI label has parallels in other settings where PPI infrastructures are still emerging [2, 3]. A recent Japanese survey of allergy patient organisations and investigators reported a similar asymmetry between patient-side and research-side actors [8], which our findings extend to individual rare disease patients and family members. These patterns are best understood in the context of how PPI has developed in Japan more broadly, as discussed below.

### PAGs as pathways to PPI awareness

The association between PAG participation and PPI awareness is one of the central findings of this study. Internationally, PAGs in rare disease research have increasingly been recognised not only as sources of peer support and information but also as partners in research collaboration. Previous studies have shown that PAGs may contribute to study design, protocol review, recruitment, research prioritisation, dissemination of research information, and broader engagement with clinical investigators [21, 22, 29]. A scoping review of patient involvement in rare disease research also suggests that patient involvement can enhance the relevance and legitimacy of research, while highlighting the practical challenges of sustaining such involvement [15].

In this context, our findings suggest that PAGs may function as important interfaces through which patients and families encounter research-related information, opportunities for involvement, and the language of PPI. The finding that PPI awareness was not significantly associated with sex, educational attainment, or patient or family member status, but was associated with PAG participation, suggests that familiarity with PPI may be shaped more by participation in organised patient communities than by individual sociodemographic characteristics. This interpretation should be made cautiously, as the cross-sectional design does not allow causal inference: people already interested in research may be more likely to participate in PAGs. Nevertheless, the result indicates that PAGs may be a key pathway for disseminating PPI-related concepts and practices in rare disease communities.

A further implication follows from the way respondents were recruited. Because participants were reached through national patient networks and were therefore, at minimum, connected to PAG channels (82.3% reported PAG participation), the finding that around 70% of the sample were nonetheless unaware of the term PPI — and that even among PAG participants roughly two-thirds (66.9%) were unaware — indicates substantial unrealised opportunity to advance PPI through patient communities. These findings suggest that patient-community channels could be used more fully to disseminate PPI-related concepts and opportunities, and point to the value of strengthening PAG outreach and capacity.

The discrepancy between awareness of terminology and self-reported experience of involvement should also be considered in this context. Some respondents reported prior experience of involvement despite not having been aware of PPI before the survey. Although the survey did not capture the specific nature of these experiences, this pattern suggests that some patients and family members may have engaged in activities broadly consistent with PPI without recognising them under the formal term. This finding cautions against equating low awareness of the term “PPI” with a complete absence of involvement. It also suggests that future studies should distinguish more carefully between awareness of PPI terminology, understanding of the concept, and experience of involvement in research-related activities.

This discrepancy may partly reflect terminological diversity. Several overlapping Japanese terms are used to describe PPI-related practices. Alongside the AMED term *kanja shimin sankaku* (患者・市民参画), these include *kanja kyōdō* (患者協働; patient engagement), *tōjisha sankaku* (当事者参画; involvement of those directly affected), and コ・プロダクション (co-production). Similar terminological overlap exists in English, where patient engagement, PPI, and co-production are related but not identical concepts. Respondents may therefore have encountered similar practices under a different label.

From an implementation perspective, these findings suggest that promoting PPI will require not only individual-level information provision but also stronger organisational capacity and outreach within PAGs, which in rare disease research often serve as the main bridge between affected families and researchers. In several international contexts, this bridging function is supported by dedicated, patient-facing training infrastructure (e.g., the European Patients’ Academy on Therapeutic Innovation (EUPATI) [30] and the European Organisation for Rare Diseases (EURORDIS) Open Academy [31] in Europe, and clinical–research collaborations such as the National Organization for Rare Disorders (NORD) Rare Disease Centers of Excellence network in the United States [22]). Although Japan has produced PPI guidance and learning resources aimed primarily at researchers [5–7], comparable patient-facing training infrastructure for rare disease PAGs and their members has not yet developed at scale. This gap is consistent with our finding that PAG participation was the only respondent characteristic examined that was associated with PPI awareness, and it suggests where additional investment may be warranted.

### Patient and family member perspectives on PPI

One recurring pattern in our data was that patients and family members tended to perceive PPI somewhat differently. Family members more often than patients identified advancement of disease research and incorporation of patient and public perspectives as benefits of PPI (Fig. 2A). They also more often identified lack of knowledge about medical research as a barrier, and educational opportunities for patients and families as a form of support they considered important. Although the specific magnitudes of these differences should be interpreted with caution given the exploratory nature of the analysis, the fact that the differences appeared consistently across benefits, barriers, and support needs suggests that patients and family members may occupy somewhat different positions in relation to PPI in rare disease research.

Family members in rare disease contexts are often deeply involved in care, information-seeking, communication with healthcare professionals, and engagement with patient organisations. Previous literature has shown that family members and carers of people with rare diseases often experience substantial psychosocial burdens and play important roles in navigating uncertainty, coordinating care, and seeking information [32–34]. Although PPI surveys rarely disaggregate responses between patients and family members, the role of family members as long-term information brokers and advocates is widely recognised in international rare disease research, and patient organisations are commonly described as the principal entry point through which patients and families encounter research opportunities [22, 32–35]. The patterns we observed — family members more strongly endorsing both the research-advancing value of PPI and the need for accessible knowledge — are consistent with these accounts and suggest that the patient–family axis may be an under-examined source of variation in international PPI work, rather than a feature peculiar to the Japanese context. Notably, family members in our sample were predominantly female (69.4%), consistent with previous caregiver studies [32, 35] and suggesting that these perspectives may also reflect gendered patterns of care and information work. These interpretations should be read as plausible accounts of the patterns we observed, given that the survey did not measure family roles or caregiving responsibilities in detail.

At the same time, these differences should not be interpreted as suggesting that family members are more suitable for PPI than patients themselves, or that patients are less interested; consistent with this, the “need to focus on treatment or health management” barrier did not differ significantly between patients (8.9% [9/101]) and family members (14.4% [16/111]; *p* = 0.215). Patients and family members occupy different positions in relation to the illness experience, and their perspectives are complementary rather than substitutable. The findings instead point to the value of differentiated and flexible approaches to involvement. Some forms of involvement may be more feasible or meaningful for patients, while others may be more appropriate for family members, depending on disease characteristics, age, disability, care responsibilities, and the purpose of the research. Rare disease PPI programmes should therefore be designed to recognise both patients and family members as having distinct but related perspectives, and to provide opportunities and support tailored to each.

### From perceived value to conditions for meaningful involvement

The findings on benefits, barriers, and support needs point to a broader practical implication. Respondents strongly endorsed advancement of disease research as a benefit of PPI, and nearly all respondents were able to identify at least one perceived benefit. Read against Abelson and colleagues’ account of the rationales for involvement [36] — developed for health technology assessment, and applied here as an interpretive lens rather than an exhaustive classification — the most frequently endorsed benefits reflect several rationales: instrumental goals, such as advancing disease research and benefiting other patients; developmental goals, such as gaining knowledge about medical research; and a more comprehensive approach through the incorporation of patient and public perspectives, which may also carry a democratic dimension insofar as it treats patient and public perspectives as relevant to research. At the same time, the most frequently selected barriers among the listed items were burden on work or daily responsibilities and lack of knowledge about medical research. The most frequently selected support needs were public information and awareness, educational opportunities for patients and families, and information in medical institutions. These findings suggest that the challenge is not simply whether rare disease patients and families see value in PPI, but whether the conditions exist to make involvement feasible, understandable, and sustainable.

This point is particularly important in rare disease research. Rare disease research often depends on sustained infrastructures such as patient registries, natural history studies, and patient-centred outcome development, all of which require long-term collaboration among researchers, clinicians, patients, families, and patient organisations [17–20]. Because rare disease communities are small, involvement may become concentrated among a limited number of active patients, family members, or PAG leaders. International reviews have identified recurring barriers to meaningful involvement, including time constraints, knowledge gaps, role ambiguity, and inadequate resources [15, 26, 27, 37]. The conditions our respondents identified — accessible public information, educational opportunities, and information provided in medical institutions — closely parallel these international syntheses, suggesting that the underlying requirements for meaningful PPI are broadly shared, even if the degree to which they are institutionally embedded varies across contexts [38, 39]. In Japan, practical initiatives such as the Commons Project and RUDY JAPAN have shown the potential of stakeholder involvement and active patient participation in rare disease research, while also indicating the importance of infrastructure, trust-building, accessible communication, and support for patient communities [24, 25]. The present findings are consistent with this literature and suggest that education, information pathways, and support for PAGs should be regarded as core conditions for meaningful PPI rather than as supplementary measures.

The high proportion of respondents selecting N/A for barriers also requires careful interpretation. It should not be read simply as indicating that barriers do not exist. Rather, it may reflect limited prior experience of involvement, difficulty imagining concrete PPI activities, or insufficient familiarity with the kinds of burdens that involvement may entail. In emerging PPI contexts, asking patients and families to identify barriers may itself presuppose a level of familiarity that many do not yet have. At the same time, the proportion selecting N/A did not differ significantly between PPI-aware and PPI-unaware respondents, so limited awareness of the term alone does not account for this pattern; for many respondents, N/A may simply indicate that none of the listed barriers applied. Where unfamiliarity is the explanation, however, information provision and educational opportunities may be prerequisites not only for participation itself but also for enabling patients and families to understand what involvement could mean. This interpretive caution is not specific to Japan: surveys conducted in any setting before patients have had structured opportunities to encounter concrete forms of involvement may underestimate the range of barriers that emerge in practice [15, 37].

### Implications for PPI policy and practice in Japan

Our findings should be understood in the context of how PPI has developed unevenly in Japan, including through disease-specific initiatives. Within this broader picture, the rare disease setting differs in important ways from more prevalent disease areas because of small and dispersed patient populations, the long-standing role of PAGs, and the heavy involvement of family members in care and advocacy. PPI awareness in our sample remained limited, yet PAG participation emerged as the only respondent characteristic examined that was associated with PPI awareness, and family members showed distinctive perception patterns. This suggests that rare disease PPI in Japan should not be understood simply as a delayed version of PPI in other fields, but as a setting in which particular actors — notably PAGs and family members — appear central to both the possibilities and the limits of involvement.

For rare disease research, our findings indicate that PPI is unlikely to develop fully through individual-level participation alone. The pattern of responses points to the importance of broader infrastructures — accessible information, educational opportunities, and support for the PAGs that mediate between patient communities and research systems — conditions also identified in international work as reducing the risk of tokenistic or overly burdensome forms of involvement [15, 26, 40]. This is particularly important as PPI becomes more visible in funding and policy frameworks. Without adequate support, researchers may draw repeatedly on a small pool of already engaged patients and family members, especially those connected to PAGs, thereby increasing the risk of overburdening them.

PPI is valued not only as a means to improve research quality but also as a way of enabling patients and the public to participate in decisions affecting their health and lives [1, 27, 37]; realising this broader potential depends on more than formal inclusion. A further implication concerns the position of Japanese rare disease patients, families, and PAGs within international research ecosystems, where the involvement of patients and their representatives has become an expected component of multinational rare disease initiatives [41–43]. The limited awareness of PPI and the perceived need for support for PAGs identified in our sample raise a practical concern that participation from Japan may meet procedural expectations for patient input without fully engaging with the patient-driven dimensions of research — a pattern characterised as tokenistic when involvement is reduced to symbolic consultation [15, 40]. Building awareness of PPI and strengthening PAG capacity within Japan are therefore important not only for domestic research but also for ensuring that patients and families in Japan are heard within international research communities.

### Limitations

Several limitations should be acknowledged. First, participants were recruited through national rare disease patient network organisations, and the findings therefore reflect the views of patients and family members who were reachable through such networks rather than a statistically representative sample. The high level of PAG participation should be interpreted in this light, and individuals without PAG connections may be under-represented. Second, although all respondents were presented with a common explanation of PPI (based on the AMED PPI Guidebook) before the relevant items, we could not verify that it was interpreted uniformly. Moreover, because the explanation preceded the awareness item, it may have influenced how respondents retrospectively judged whether they had previously encountered PPI. Self-reported prior awareness and experience, which refer to the period before the survey, should therefore be interpreted with some caution. In addition, because the introductory explanation described the purposes and examples of PPI in largely positive terms, it may have framed respondents’ subsequent assessments of its benefits, barriers, and support needs. Third, the cross-sectional design precludes causal inference: it is not possible to determine whether PAG participation leads to PPI awareness, whether people already interested in research are more likely to join PAGs, or whether both are influenced by other factors such as health literacy or prior research experience.

Fourth, the sample size was modest and subgroup analyses were exploratory; multiple comparisons were not formally adjusted, and the findings on individual items — particularly the patterns observed between patients and family members — are best regarded as hypotheses to be confirmed in larger studies. The category of “family members” may also encompass heterogeneous roles (e.g., parents of affected children, adult carers, siblings, and partners), which this study could not examine separately. In addition, family members in the sample were predominantly female. Finally, the survey used predefined response options without an open-ended (“other, please specify”) field, so respondents could not record considerations outside the listed categories; moreover, selecting N/A indicates only that none of the listed options was chosen and reflects our interpretation of “none of the listed items applied” rather than a verified absence of relevant benefits, barriers, or support needs. Future qualitative research would help deepen understanding of how PPI is experienced and supported in practice.

Despite these limitations, this study provides empirical evidence on how rare disease patients and family members in Japan perceive PPI at a time when institutional interest in PPI is increasing.

## Conclusions

This study provides empirical evidence on awareness, perceived benefits and barriers, and support needs regarding PPI among rare disease patients and family members in Japan. Although prior awareness of PPI was limited, participation in PAG activities was associated with PPI awareness, suggesting that organised patient communities may play an important role in connecting patients and families with research-related information and opportunities for involvement. Respondents generally recognised the value of PPI, particularly for advancing disease research, while also identifying the need for public information, educational opportunities, and information in medical institutions as conditions for involvement. Notably, family members differed from patients across multiple domains: they more often identified research advancement and the incorporation of patient and public perspectives as benefits, more frequently identified knowledge gaps as barriers, and more strongly endorsed educational opportunities as a needed form of support. These patterns suggest that patients and family members may occupy somewhat different positions in rare disease PPI and may require differentiated forms of engagement and support. Overall, meaningful PPI in rare disease research requires more than formal promotion; it depends on practical conditions that allow patients, families, and PAGs to contribute in ways that are feasible, supported, and sustainable.

## Supplementary Information

Below is the link to the electronic supplementary material.

Supplementary Material 1: Additional file 1. GRIPP2 Checklist (DOCX). Completed GRIPP2 short form checklist.

Supplementary Material 2: Additional file 2. Survey Questionnaire (DOCX). English translation of the questionnaire administered in Japanese, including the introductory explanation of PPI presented before the awareness item.

## Data Availability

The datasets used and analysed during the current study are available from the corresponding author on reasonable request, subject to appropriate consideration of participant privacy and the conditions under which the data were collected. The questionnaire and dataset were developed in Japanese.

## Abbreviations

AMED: Japan Agency for Medical Research and Development
EUPATI: European Patients’ Academy on Therapeutic Innovation
EURORDIS: European Organisation for Rare Diseases
GRIPP2: Guidance for Reporting Involvement of Patients and the Public 2
N/A: Not applicable (none of the listed options applied)
NORD: National Organization for Rare Disorders
PAG: Patient advocacy group
PPI: Patient and public involvement
