# Supplementary material for: Awareness and perceptions of patient and public involvement in rare disease research in Japan: the role of patient advocacy groups and family members
Source: Res Involv Engagem. 2026 Aug 13;12:125. doi: 10.1186/s40900-026-00955-9 (PMC13474695; doi:10.1186/s40900-026-00955-9)
Supplement: Supplementary file 1 [file 40900_2026_955_MOESM1_ESM.docx]

**GRIPP2 Short Form Checklist**

Guidance for Reporting Involvement of Patients and the Public – Version 2 (Short Form)

**Manuscript title:** Awareness and perceptions of patient and public involvement in rare disease research in Japan: the role of patient advocacy groups and family members

**Reference:** Staniszewska S, Brett J, Simera I, Seers K, Mockford C, Goodlad S, et al. GRIPP2 reporting checklists: tools to improve reporting of patient and public involvement in research. Research Involvement and Engagement. 2017;3:13.

**Note:** This checklist uses the GRIPP2 Short Form because patient and public input in this study was limited to questionnaire development and recruitment support, rather than full co-production across all stages of the research process. PPI, patient and public involvement; PAG, patient advocacy group.

| **Section and topic** | **Item** | **Reported information** |
| --- | --- | --- |
| **1. Aim** | Report the aim of PPI in the study. | Patient and public input was sought to improve the clarity, accessibility, and acceptability of the survey materials for rare disease patients and family members. The broader study aimed to explore awareness, prior experience, perceived benefits and barriers, and support needs regarding PPI among rare disease patients and family members in Japan. |
| **2. Methods** | Provide a clear description of the methods used for PPI in the study. | Representatives of two cooperating rare disease patient network organisations, the Japan Patients Association and the Support Network for Nanbyo Children of Japan, provided input during questionnaire development. They reviewed and pilot-tested the draft questionnaire by completing the survey and giving feedback on items that were unclear or potentially difficult or distressing for patients and family members. They also provided advice on the wording and format of the questionnaire and invitation materials. Their feedback was used to revise the survey materials before implementation. |
| **3. Study results** | Report the results of PPI in the study, including both positive and negative outcomes. | Feedback from the cooperating patient network organisations led the research team to revise the wording and structure of the questionnaire and invitation materials. The cooperating organisations also facilitated recruitment through their national rare disease patient networks. The effects of the PPI process were not formally evaluated, and no negative effects were identified. However, PPI was limited to the questionnaire development and recruitment stages; patients and members of the public were not involved in data analysis or manuscript drafting. |
| **4. Discussion and conclusions** | Comment on the extent to which PPI influenced the study overall. Describe positive and negative effects. | The involvement of patient network organisations contributed to making the survey more accessible and appropriate for potential respondents. At the same time, recruitment through these networks shaped the study sample, which included a high proportion of respondents with prior PAG participation. This should be considered when interpreting the findings, as the sample reflects patients and family members reachable through national patient networks rather than all rare disease patients and families in Japan. |
| **5. Reflections / critical perspective** | Comment critically on the PPI process, including what worked well and what could be improved. | The PPI input was useful for improving the survey materials, but the involvement was limited in scope and did not extend across the full research cycle. Future research would benefit from involving rare disease patients and family members more extensively across the research process, including study design, interpretation of findings, dissemination, and the development of recommendations for PPI practice. An accessible summary of the findings will be shared with the two cooperating patient network organisations after publication. |
