# Supplementary material for: Awareness and perceptions of patient and public involvement in rare disease research in Japan: the role of patient advocacy groups and family members
Source: Res Involv Engagem. 2026 Aug 13;12:125. doi: 10.1186/s40900-026-00955-9 (PMC13474695; doi:10.1186/s40900-026-00955-9)
Supplement: Supplementary file 2 [file 40900_2026_955_MOESM2_ESM.docx]

**Additional file 2**

**Survey questionnaire**

*English translation of the survey questionnaire originally administered in Japanese.*

The questionnaire was developed in Japanese and administered via a web-based survey platform. For items B1, C1, and D1, respondents were asked to select all applicable options. Items have been translated into English for the purpose of this publication; item order and wording have been preserved as closely as possible.

Introductory explanation of PPI presented to all respondents before the awareness item (Section A). This explanation was based on the definition and examples in the AMED Patient and Public Involvement (PPI) Guidebook.

*[Heading shown to respondents: “Questions about patient and public involvement”]*

In this survey, patient and public involvement (PPI) refers to efforts to make medical research and clinical trials safer and more grounded in patients’ perspectives by incorporating the voices and perspectives of patients and members of the public into the research process.

PPI takes many forms and operates at many levels — for example, seeking the opinions of patients and members of the public from the outset when deciding how a study should be conducted; seeking their opinions when preparing informed-consent documents; and asking patient associations to help recruit research samples and participants.

**Questionnaire items**

| **Item** | **Response options** |
| --- | --- |
| **Section A. Awareness and experience of PPI** | |
| A1. Before taking this survey, had you heard of patient and public involvement (PPI)? | - Yes - No |
| A2. Have you previously been involved in PPI-related activities in medical research or drug development? | - Yes - No |
| **Section B. Perceived benefits of PPI** | |
| B1. In your view, what are the potential benefits of taking part in PPI? (Select all that apply.) | 1. Advancement of disease research  2. Acquiring knowledge about medical research  3. Contributing to the benefit of other patients  4. Incorporation of patient and public perspectives into research  5. Contribution to society through patient and public engagement  6. Receiving financial reward for participation  7. None of the above applies (N/A) |
| **Section C. Perceived barriers to involvement in PPI** | |
| C1. In your view, what are the potential barriers to taking part in PPI? (Select all that apply.) | 1. Lack of knowledge about medical research among patients and the public  2. Need to focus on treatment or health management  3. Limited expected impact of PPI on research outcomes  4. Burden on work or daily responsibilities  5. Anxiety about communication with healthcare professionals and researchers  6. Limited financial compensation for participation  7. None of the above applies (N/A) |
| **Section D. Support needs for promoting PPI** | |
| D1. What kinds of support do you think are needed to promote PPI among patients and their families? (Select all that apply.) | 1. Educational opportunities for patients and families  2. Educational opportunities for patient advocacy group (PAG) leaders  3. Public information and awareness about PPI  4. Information provided in medical institutions  5. Financial support for PAGs  6. Personal reward or compensation for PPI contributors  7. None of the above applies (N/A) |
| **Section E. Respondent characteristics** | |
| E1. Sex | - Male - Female |
| E2. Year of birth | Free entry; converted to age and grouped for analysis as <20, 20–29, 30–39, 40–49, 50–59, 60–69, ≥70 |
| E3. Highest educational attainment | - Junior high school - Senior high school - Technical or vocational school - Junior college - University - Graduate school |
| E4. Respondent type | - Person living with a rare disease (patient) - Family member of a person with a rare disease - Other (e.g., supporter, healthcare professional, or member of the public not personally affected by a rare disease) |
| E5. Have you participated in patient advocacy group (PAG) activities? | - Yes - No |

Notes: PPI, patient and public involvement; PAG, patient advocacy group. Response options for items B1, C1, and D1 have been reordered in the published figures based on the proportion of respondents selecting each option; the option codes given above reflect the original presentation. Respondents who selected ‘Other’ for E4 (n = 6) were excluded from the analysis as they did not meet the eligibility criteria of being a person living with a rare disease or a family member of such an individual.
